# Advances in second hematopoietic stem cell transplantation

**DOI:** 10.3389/fimmu.2024.1428101

**Published:** 2024-07-09

**Authors:** Sijia Yan, Xiaojian Zhu, Yi Xiao

**Affiliations:** Department of Hematology, Tongji Hospital, Tongji Medical College, Huazhong University of Science and Technology, Wuhan, China

**Keywords:** second hematopoietic stem cell transplantation, relapse, prognostic factor, hematological malignancy, first hematopoietic stem cell transplantation

## Abstract

Hematopoietic stem cell transplantation (HSCT) is a widely used treatment for malignant hematological diseases; however, some patients inevitably experience relapse. Therefore, for patients who relapse after the first HSCT (HSCT1), a standard treatment regimen must be developed. A second hematopoietic stem cell transplantation (HSCT2) is a possible treatment option. Several studies have analyzed the feasibility of HSCT2. Previous studies have shown that various factors may affect the efficacy of HSCT2, including the hematopoietic cell transplantation comorbidity index, duration of remission after HSCT1, occurrence of chronic graft-versus-host disease, and disease status before HSCT2. However, the selection of donors for HSCT2 does not affect the transplantation efficacy. HSCT2 also presents a risk of relapse, and the prognosis of patients after relapse is poor. Further research on the treatment of patients after relapse is warranted.

## 1 Introduction

Hematopoietic stem cell transplantation (HSCT) is a widely used therapy for hematological malignant diseases; however, approximately 30–40% of patients inevitably relapse (1). There is no standard treatment for patients who relapse after the first HSCT (HSCT1). A second HSCT (HSCT2) may be one possible way to solve this problem. Although HSCT2 does not lead to sustained remission, a considerable proportion of patients remain disease-free for up to a year after HSCT2, providing opportunities for other treatments (2). Multiple factors influence the prognosis of patients undergoing HSCT2 (3).

## 2 Feasibility of HSCT2

HSCT2 and donor lymphocyte infusion (DLI) are commonly used as salvage treatments for relapse after HSCT1. The study of Al-Shaibani et al. compared the effects of two cell therapies, HSCT2 and DLI, on the outcomes of patients with HSCT1 graft failure or relapse. They suggest that HSCT2 may improve the prognosis of patients who relapse after HSCT1 if they go into remission again, whereas DLI is suitable for patients with active disease (4). The results of several studies (**Table 1**) have also confirmed that HSCT2 is an effective and feasible treatment option for patients after graft failure or relapse after the first transplantation, which can bring new hope to some patients.

**Table 1:** Feasibility analysis.

Trial	N	Indication for HSCT2	OS	NRM/TRM	CIR	LFS/DFS
Shumilov et al.	30	relapse	3-year, 43%	33%	30%	3-year LFS, 33%
Filippini et al.	82	relapse	2-year, 34.3%	2-year, 17.6%	2-year, 57%	2-year LFS, 25.4%
Panz-Klapuch et al.	18	relapse	2-year, 61%	2-year, 17%	2-year, 22%	/
Jaime‐Pérez et al.	ASCT1, 12	relapse	1-year, 66.7%	2-year, 30%	/	1-year DFS, 59%
	allo-HSCT1, 15	12 relapse, 3 graft failure	1-year, 43.3%		/	1-year DFS, 36%
Ma et al.	30	Graft failure	1-year, 60%	1-year, 33.3%	1-year, 6.7%	1-year DFS, 53.3%
Sun et al.	13	Graft failure	1-year, 56.6%	1-year, 33.3%	/	1-year DFS, 48.4%
Gyurkocza et al.	126	Relapse	2-year, 33%	2-year, 33%	2-year, 42%	/
Choi et al.	80	Relapse	2-year, 21%	2-year, 18.7%	2-year, 60.2%	2-year, 17.7%
Zhao et al.	44	Relapse	1-year, 80.6%	1-year, 2.3%	1-year, 25.1%	1-year, 72.5%
Yalniz et al.	91	Relapse	2-year, 36%	2-year, 18%	2-year, 42%	2-year, 27%
Lu et al.	199	Relapse	2-year, 43.8%	2-year, 38.5%	2-year, 30%	2-year, 42.1%
Srour et al.	29	Relapse	3-year, 40%	3-year, 39%	3-year, 30%	3-year, 31%

HSCT2, second hematopoietic stem cell transplantation; ASCT1, first autogenic hematopoietic stem cell transplantation; allo-HSCT1, first allogenic hematopoietic stem cell transplantation; OS, overall survival; NRM, non-relapse relative morbidity; CIR, cumulative incidence of relapse; LFS, leukemia-free disease; DFS, disease-free survival; TRM, transplant-related mortality.

### 2.1 HSCT2 in patients who relapse after HSCT1

Shumilov et al. studied 123 newly diagnosed acute myelogenous leukemia (AML) patients receiving high-dose chemotherapy (HDCT)/autogenic stem cell transplantation (auto-HSCT, ASCT) after the first complete remission (CR), and analyzed the results of salvage therapy (5). Of the 123 patients, 64 (52%) experienced relapse after ASCT. Subsequently, 13 patients (21%) received palliative care, and 51 (79%) received salvage care. Finally, 30 patients (47%) underwent allogeneic hematopoietic stem cell transplantation (allo-HSCT). After allo-HSCT2, 19 patients (63%) achieved CR, and relapse or progression occurred in 11 patients (37%) after allo-HSCT2. At the last follow-up, 11 patients (37%) were alive and in remission, 10 (33%) died of non-relapse relative morbidity (NRM) during CR, and 9 (30%) died of disease progression. The estimated 3-year overall survival (OS) and leukemia-free survival (LFS) were 43% and 33%, respectively. Their findings suggest that most patients with AML who relapse after first-line HDCT/ASCT can receive salvage therapy, and allo-HSCT is an effective salvage method for patients with relapse after HSCT1.

Panz-Klapuch et al. focused on patients with Ph-negative acute B lymphocytic leukemia (B-ALL) with relapse after auto-HSCT1 (6). This study retrospectively analyzed 18 patients. After allo-HSCT2, the 2-year cumulative incidence of relapse (CIR) and NRM rates were 22% and 17%, respectively. The 2-year OS was 61%. At the last follow-up, five patients were alive and remained in CR with full donor chimerism. In patients with acute leukemia (AL), allo-HSCT2 after auto-HSCT1 failure is a viable treatment option and is associated with a relatively high proportion of patients being cured.

Velazquez et al. conducted a retrospective analysis of adults with AL, including 82 patients who underwent HID-HSCT2 after a hematologic relapse of HID-HSCT1 (7). The median follow-up duration was 33 months. The 2-year OS, NRM, and CIR rates were 34.3%, 17.6%, and 57%, respectively. This study included the largest cohort of dual HID-HSCT patients analyzed to date, and the data show the feasibility of a second HID-HSCT after HID-HSCT1 relapse, with a high implantation rate and acceptable NRM. HID-HSCT2 is a viable option for patients with AL who experience relapse after HID-HSCT1. The toxicity, risk factors, and long-term outcomes of HID-HSCT2 are similar to those of MUD-HSCT2.

Jaime-Perez et al. suggested that allo-HSCT2 is a viable alternative for patients with severe life-threatening blood diseases after auto/allo-HSCT failure or relapse (8). Their retrospective study included 27 patients, of whom 12 underwent auto-HSCT1 (all relapsed) and 15 underwent allo-HSCT1 (including 12 relapsed and 3 transplant failures). The 1-year OS and disease-free survival (DFS) in the auto-HSCT1 group were 66.7% and 59%, respectively. In the allo-HSCT1 group, the 1-year OS and DFS rates were 43.3% and 36%, respectively, whereas the 2-year OS rate was 26%. However, the sample size of this study was small. Future studies with a larger sample size are needed.

### 2.2 HSCT2 in patients with graft failure after HSCT1

Second transplantation is also a viable treatment option for patients with graft failure. Ma et al. used a new conditioning regimen, namely fludarabine combined with cyclophosphamide, to perform haplo-identical donor-HSCT2 (HID-HSCT2) from different donors (9). Their study included 30 patients in whom the first transplantation failed and who received a salvage transplant with a new conditioning regimen. At the time of HSCT2, 16 (53.3%) patients had complete donor chimerism, 8 (26.7%) had mixed chimerism, and 6 (20%) had complete recipient chimerism. In total, 20 patients survived at a median follow-up period of 295 days. The 1-year OS and LFS rates were 60% and 53.3%, respectively. The CIR and transplant-related mortality (TRM) were 6.7% and 33.3%, respectively. The 1-year OS of the new regimen group also significantly improved compared to that of the other historical conditioning regimens (60.0% vs. 26.4%, P=0.011). In conclusion, HID-HSCT2 with a different donor following a low-toxicity preconditioning regimen is a promising option for saving patients with GF after HID-HSCT1.

HID-HSCT is increasingly used for AL patients who do not have HLA-matched siblings (sibs) or matched unrelated donors (MUDs). A prospective trial in China (NCT03717545) (10) included 13 patients who had failed their HSCT1. At HSCT1, 11 patients underwent HID-HSCT, and 2 received MUD-HSCT. At the time of transplant failure, 3 patients had complete recipient chimerism, 5 had mixed chimerism, and 5 had complete donor chimerism. At HSCT2, all 13 patients received HSCT2 from different HIDs, and the 1-year TRM, OS, and DFS were 23.1%, 56.6%, and 48.4%, respectively. The results of this study suggest that HSCT2 with a different HID is a promising salvage option for patients who experience a relapse after HSCT1. However, the trial included a small number of participants, and further studies are required to confirm the applicability of this approach.

The results of several studies have proven that HSCT2 is an effective salvage method for patients with hematological malignant diseases, regardless of the donor selected for the first transplant, after first transplant failure or relapse, and can significantly improve the prognosis of some patients, bringing new hope.

## 3 Factors affecting the efficacy of HSCT2

HSCT2 may also be subject to relapse. Multiple factors may influence the prognosis of patients with HSCT2, and several studies are currently being explored (**Table 2**). At present, the objects of most relevant research are AL patients.

**Table 2:** Analysis of prognostic factors for HSCT2.

	N	Subgroups	2-year OS	P-value	2-year DFS/PFS	P-value	2-year CIR	P-value	2-year NRM	P-value
Lee et al.	50	HID-HSCT2, 31	41%	/	41%	/	36%	/	23%	/
		CBT, 19	29%		16%		36%		48%	
Shimoni et al.	556	Same donor, 163	36.4%	0.21	/	/	51.5%	0.90	25.1%	0.28
		Different matched donor, 305	28.7%				49.3%		26.9%	
		HID, 88	23.3%				44.2%		33.9%	
Kharfan et al.	455	MUD, 320	31%	0.57	25%	0.73	56%	0.44	26%	0.53
		HID, 135	29%		29%		48%		27%	
Gyurkocza et al.	126	/	33%	/	/	/	42%	/	33%	/
Choi et al.	80	Group 1, 36	38.3%	<0.001	30.6%	<0.001	58.3%	0.034	11.1%	0.185
		Group 2, 30	10%		10%		60%		26.7%	
		Group 3, 14	0%		0%		64.3%		21.4%	
Zhao et al.	44	CR, 33	1-year, 81.8%	/	1-year, 79.9%	/	1-year, 16.8%	/	2.3%	/
		NR, 11	1-year, 76.2%		1-year, 51.9%		1-year, 48.1%			
Han et al.	28	-	1-year, 25%	/	1-year, 21.4%	/	53.5%	/	1-year, 25%	/
Yalniz et al.	91	-	36%	/	27%	/	44%	/	18%	/
Lu et al.	199	-	43.8%	/	42.1%	/	30%	/	38.5%	/
Srour et al.	29	-	3-year, 40%	/	3-year, 31%	/	3-year, 30%	/	3-year, 39%	/
Hou et al.	24	-	46%	/	37.5%	/	40%	/	1-year, 29%	/
Hayden et al.	215	Relapse, 159	38%	/	17%	/	68%	/	15%	/
		Graft failure, 56	41%		34%		32%		34%	

Group 1:CR/CRi at HSCT2 and remission duration ≥6 months after HSCT1.

Group 2: Active disease at HSCT2 or remission duration <6 months after HSCT1.

Group 3: Active disease at HSCT2 and remission duration <6 months after HSCT1.

OS, overall survival; DFS, disease-free survival; PFS, progression-free survival; CIR, cumulative incidence of relapse; NRM, non-relapse mortality; HID, haploidentical donor; MUD, matched unrelated donor; CBT, cord blood transplantation; CR, complete remission; CRi, CR with incomplete count recovery; NR, no response; HSCT1, first hematopoietic stem cell transplantation; HSCT2, second hematopoietic stem cell transplantation.

### 3.1 Selection of donors

Cord blood stem cells serve as an alternative donor source. Lee et al. compared the results of patients with relapsed AML after HSCT1 receiving replacement donors for HSCT2 (11): a total of 50 AML patients were included in their study, with 31 patients and 19 patients receiving HID-HSCT and cord blood transplantation (CBT), respectively. At a median follow-up of 64.6 months, the 2-year OS was 41% and 29%, 2-year DFS was 41% and 16%, 2-year CIR was 36% and 36%, and 2-year NRM was 23% and 48%, respectively, in the HID-HSCT and CBT groups. The results showed that HID-HSCT resulted in a better prognosis than CBT.

Previous researchers have speculated that donor replacement in HSCT2 may result in a new graft-versus-tumor effect that enhances efficacy; however, the results of several studies contradict this idea. The Acute Leukemia Working Party (ALWP) of European Society for Blood and Marrow Transplant (EBMT) has also studied allo-HSCT2 donor selection for AML patients who relapse after HSCT1 (12). There are three donor types in HSCT2, including MUD, sib, and HID. The researchers retrospectively analyzed the results of HSCT2 in 556 patients divided into three groups: Same donor (n=163, sib/sib-112, MUD/MUD-51), different matched donors (n=305, sib/different sib-44, sib/MUD-93, MUD/different MUD-168), or HID (n=88, MUD/different MUD-168, sib/HID-45, MUD/HID-43). In the same donor group, different matched donor group, and haploid matched donor group, the 2-year NRM was 25.1%, 26.9%, and 33.9% (P=0.28); the 2-year CIR was 51.5%, 49.3%, and 44.2% (P=0.90); and the 2-year OS was 36.4%, 28.7%, and 23.3% (P=0.21), respectively. In summary, HSCT2 using the same or different matched donors resulted in similar outcomes for patients with relapsed AML.

Kharfan et al. studied the effect of allo-HSCT2 from different donors on the survival rate of relapsed AML (13), a total of 455 patients with relapsed AML were included in this study, among whom 320 received allo-HSCT2 from MUDs and 135 received allo-HSCT2 from HIDs. The median follow-up from allo-HSCT2 was 30 months. For patients with active AML receiving allo-HSCT2, the CR rate (CRR) after allo-HSCT2 was similar in the MUD and HID groups (63% vs. 60%, respectively, P=0.72).

Gyurkocza et al. analyzed the results of 126 patients with hematological malignancies and also proved that donor replacement had no significant impact on patients receiving HSCT2 (14). These patients relapsed after the first allo-HSCT and received a second allo-HSCT. A total of 17 patients used the original donors and 109 patients used different donors. The 2-year OS, relapse rate, and NRM were 33%, 42%, and 33%, respectively. The results showed that HSCT2 with a different donor from HSCT1 did not adversely affect the results.

### 3.2 Duration of remission after HSCT1 and status before HSCT2

As stated by Kharfan et al., multivariate analysis showed that the presence of active AML at allo-HSCT2 correlated with lower LFS OS, CIR, and graft-versus-host disease (GVHD)- and relapse-free survival (GRFS). The longer the duration from allo-HSCT1 to relapse (>14.5 months), the higher the OS, LFS, and GRFS and the lower the RI and NRM. Additionally, the older the patient, the lower the OS. Their study emphasized that allo-HSCT2 in CR achieved the best OS (13). Gyurkocza et al. showed that patients with early relapse (<12 months) after HSCT1 had a higher relapse rate and worse OS. For patients who relapse after the first transplantation, especially those who relapse more than one year later, a second allo-HSCT should be considered (14).

Choi et al. concluded that disease status at HSCT2 (active disease and CR/CR with CRi) and the duration of remission after HSCT1 are important factors affecting the prognosis of HSCT2 (15). They divided AML patients who relapsed after HSCT1 into three groups according to two prognostic factors. There were 36 patients in group 1 (CR/CRi at HSCT2 and remission duration after HSCT1 ≥ 6 months) and 30 patients in group 2 (active disease at HSCT2 or remission duration after HSCT1 < 6 months). Group 3 (active disease at HSCT2 and remission duration of <6 months after HSCT1) included 14 patients. This conclusion is based on the fact that patients in group 1 had a 2-year OS of 38.3%, whereas patients in group 3 did not survive for more than 1 year after transplantation and had a median OS of only 1 month.

A Chinese study showed that disease status before HSCT2 is the most important factor affecting the efficacy of HSCT2 (16). A total of 44 patients with relapse after HSCT1 were included in this study, and the disease status before transplantation was CR in 33 cases (75%), of which 26 cases (59.1%) were MRD-negative, 7 cases (15.9%) were MRD-positive, and no response (NR) was observed in 11 patients (25%). By the end of follow-up, 10 patients had relapsed completely, and 5 patients in the CR and NR groups had relapsed before transplantation. At a median follow-up of 14 months, the 1-year OS and DFS rates were 80.6% and 72.5%, respectively, with a CIR of 25.1%. The OS and CIR rates were 81.8% and 16.8%, respectively, in the CR group and 76.2% and 48.1% in the NR group, respectively. Univariate analysis revealed that whether the primary disease was in CR before secondary transplantation was an important prognostic factor.

Currently, there are few studies on the efficacy of HSCT2 in patients with relapsed/refractory AL treated with post-chemotherapy plus modified donor lymphocyte infusion (post-chemotherapy + m-DLI) after HSCT1. Therefore, one retrospective study analyzed the efficacy of HSCT2 in 28 patients with relapsed/refractory AL after chemotherapy and m-DLI (17). At a median follow-up of 918 days, 26 patients (92.9%) achieved CR, and 2 patients presented with persistent disease. One year after HSCT2, the OS and DFS rates were 25.0% and 21.4%, respectively. The NRM on day 100 and 1 year after HSCT2 was 7.1% and 25.0%, respectively. The CIRs at 1 and 2 years after HSCT2 were 50.0% and 53.5%, respectively. The data showed that relapse within six months of HSCT1 was an independent risk factor for relapse and DFS after HSCT2.

### 3.3 Chronic graft-versus-host disease

cGVHD after HSCT1 also affects the prognosis of patients after HSCT2. Yalniz et al. retrospectively analyzed 91 patients with relapsed AML treated with HSCT2 between 2000 and 2019 (18), with a median age of 44 years. In total, 53% of patients had a different donor at HSCT2, and 61% were in remission at HSCT2. The median duration of remission after HSCT1 was 8.4 months, and the median duration between transplants was 14 months. The median follow-up time for HSCT2 was 66 months, and the 2-year OS, progression-free survival (PFS), and NRM rates were 36%, 27%, and 18%, respectively. Multivariate analysis showed that low OS after HSCT2 was associated with cGVHD after HSCT1 and hematopoietic cell transplantation comorbidity index (HCT-CI) ≥2 at HSCT2 (HR=2.9 and 2.6, respectively). Their research proved that HSCT2 is feasible for AML patients who relapse after HSCT1 treatment. Long-term survival benefits are achievable in patients without cGVHD and with an HCT-CI <2 at HSCT2. Previous researchers have suggested that the failure of GVHD-mediated graft-versus-leukemia (GVL) activity after the first transplant is an important factor leading to relapse after HSCT1. However, few studies have elucidated the mechanisms by which this effect occurs, and further exploration is needed (19).

### 3.4 HCT-CI

HCT-CI has been widely used to evaluate preexisting comorbidities before transplantation, which can predict patient prognosis. A high HCT-CI is closely related to low OS (20). Lu et al. proposed a new multivariate analysis of pretransplant factors (21). They included CR/MRD-negative disease status, HCT-CI score prior to allo-HSCT2, and new HID at HSCT2 as the prognostic factors that, when present, may lead to better OS and leukemia-free survival (LFS) in patients with hematological malignancies receiving allo-HSCT2. Their results showed that patients with all three positive prognostic factors exhibited higher 2-year OS and LFS and lower CIR and TRM; patients without any of these positive prognostic factors had lower 2-year OS and LFS and higher CIR and TRM.

Srour et al. confirmed the influence of HCT-CI on patient prognosis after HSCT2 (22). They included 29 patients with hematologic malignancies who relapsed after allo-HSCT1 and underwent HID-HSCT2; only 24% achieved CR at HSCT2. The median HCI-CI score before HSCT2 was 2, while the scores of 11 patients (37.9%) were ≥3. The median follow-up was 46.9 months, and the 3-year CIR, NRM, PFS, and OS rates were 30%, 39%, 31%, and 40%, respectively. In multivariate analysis, a higher HCT-CI was strongly associated with lower OS (HR=1.32), PFS (HR=1.32), and NRM (HR=1.30), whereas patients with a lower HCT-CI receiving HSCT2 had better survival rates.

### 3.5 Conditioning regime

At present, the commonly used conditioning regimes before transplantation are divided into myeloablative conditioning (MAC) and reduce-intensity conditioning (RIC). Choi et al. analyzed the effects of two conditioning regimes on the efficacy of HSCT2 (15). Of the 60 patients who received HSCT2, 13 received MAC and 67 received RIC. The 2-year OS and EFS were 38.5% and 17.8% (P=0.418), respectively, and 38.5% and 13.4% (P=0.279), respectively, showing no statistical difference. Srour et al. also analyzed these two conditioning regimes (22). In their study, 15 patients received MAC and 14 patients received RIC. After the HSCT2, the 3-year PFS for both were 24% and 40%, and for OS 40% and 39.7%, respectively. Similar to the results of Choi et al., the study of Srour et al. showed no statistical difference in the efficacy of conditioning regimes for HSCT2. Therefore, the choice of conditioning regimes may have no significant effect on the efficacy of HSCT2.

However, the Beijing protocol (immunosuppression based on anti-thymoglobulin and granulocyte colony-stimulating factor) as a kind of MAC, while showing its advantages in the HID-HSCT1, may also be one of the prognostic factors in HSCT2. A study from the First Affiliated Hospital of Soochow University included 24 patients who relapsed or failed after HSCT1 to undergo HSCT2 (23): HSCT1, 17 patients (70.8%) received auto-HSCT, and 7 patients (29.2%) received allo-HSCT. Twenty patients (83.3%) relapsed after HSCT1, and four patients (16.7%) developed graft failure after HSCT1. The median interval between transplants was 288 days. Before HSCT2, 14 patients (58.3%) had active disease, 6 (25%) had CR, and 4 (16.7%) experienced graft failure. At HSCT2, 22 patients received the Beijing protocol. The median follow-up time was 416 days, and 16 patients (66.7%) died during the follow-up. The 2-year PFS of HSCT2 was 37.5%, OS was 46%, and 1-year NRM was 29%, which were better than those of the secondary HID-HSCT with the post-transplant cyclosporine regimen. At HSCT1, 17 patients received auto-HSCT and 7 patients received allo-HSCT; there was no significant difference in the 2-year PFS (29% vs. 35%) and OS (43% vs. 48%) between the allo-HSCT and auto-HSCT groups.

The results of existing research show that for AL patients who relapse after HSCT1, replacing a donor different from that in HSCT1 at HSCT2 has no influence on the outcome. A sib is the preferred donor for HSCT2. HID or cord blood can also be used as a donor for HSCT2 in cases where sibling donors are unavailable; however, the superiority of the two remains uncertain.

HLA loss is a common cause of relapse after HSCT1. Approximately 10-30% of relapses following HLA-mismatched HSCT involve the loss of receptor-specific HLA genes, thereby rendering leukemia cells resistant to GvL effects (24). In the case of receptor-specific HLA loss, the use of alloreactive T cells, such as donor lymphocyte infusion (DLI), to control relapse after HSCT would be ineffective and would increase the risk of GvHD. Therefore, it is necessary to detect HLA after HLA mismatch HSCT, which can help to better select donors of HSCT2.

HCT-CI prior to allo-HSCT2, duration of remission after HSCT1, occurrence of cGVHD, and disease status before HSCT2 were all important factors affecting the efficacy of HSCT2. Patients with high HCT-CI scores before HSCT2, a short duration of remission after HSCT1, early relapse, cGVHD, and pre-HSCT2 active disease had lower OS and higher relapse rates. Additionally, the use of MAC before HSCT2 did not significantly advance the efficacy of the HSCT2, but the Beijing regimen of MAC may be due to PTCY regimen. However, most of the existing studies are retrospective, and the sample size of randomized controlled trials is very small; therefore, more samples and longer follow-ups are still needed for verification. Therefore, further studies are warranted in this regard.

**Figure 1:**
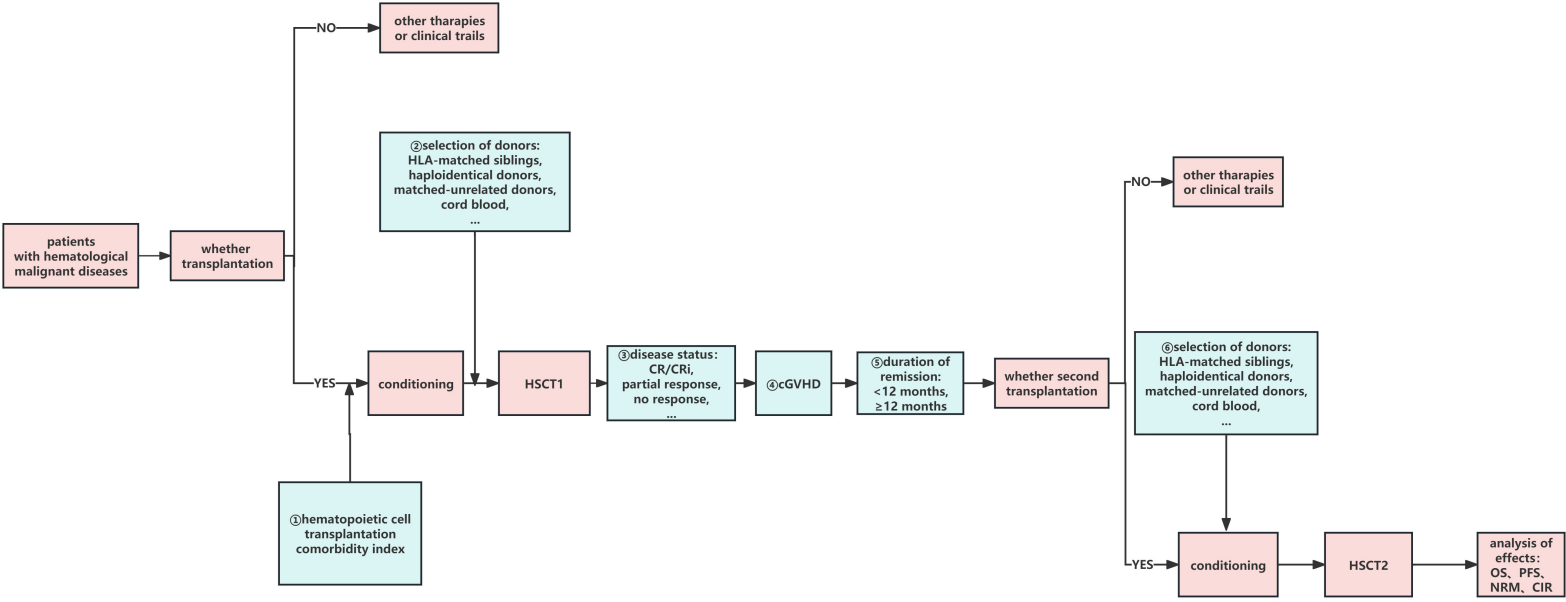
Factors influencing HSCT2. ①–➅:possible factors of HSCT2 which may affect the curative effect. HSCT1, first hematopoietic stem cell transplantation; HSCT2, second hematopoietic stem cell transplantation; CR, complete remission; CRi, CR with incomplete count recovery; cGVHD, chronic graft-versus-host disease; OS, overall survival; DFS, disease-free survival; PFS, progression-free survival; CIR, cumulative incidence of relapse; NRM, non-relapse mortality.

At present, most studies related to second transplantations have focused on AL but few have focused on multiple myeloma (MM). The European Society for Blood and Marrow Transplantation (EBMT) Chronic Malignancies Working Party conducted a retrospective analysis of 215 patients who underwent allo-HSCT2 for MM (25), among whom 159 had relapse and 56 had graft failure. In the relapse group, the 2-year OS and PFS rates were 38% and 17%, respectively, and the 5-year OS and PFS rates were 25% and 6%, respectively. The 5-year OS was significantly better when sibs were used for both transplants than that with other donor types (35% vs. 9%); patients who underwent allo-HSCT2 at 2 years also had significantly decreased OS and PFS compared to patients who underwent re-transplantation after 2 years: 10% OS at 2 years versus 31% after 5 years, and 9% PFS versus 20% PFS, respectively. Among patients with advanced disease, 2-year CIR was significantly higher than that of patients with low disease (77% vs. 57%). In contrast, in patients with a low disease burden, NRM was significantly higher at 2 years (25% vs. 11%), older age at allo-HSCT2 (50–70 vs. <50 years) was associated with higher CIR at 2 years (77% vs. 56%), and cumulative NRM at 2 years was significantly higher in younger individuals (24% vs. 9%). In the graft failure group, the 2-year OS and PFS rates were 41%, 34%, respectively. Therefore, for patients with MM who relapse after HSCT1, a second transplantation with sibs is one possible way to improve the efficacy of HSCT2. The longer the interval between transplants, the lower the disease burden, and the younger the age at the second transplant, the better the patient prognosis (**Figure 1**).

## 4 Relapse after HSCT2

At present, there are few studies on maintenance therapy after secondary transplantation in AL patients. As for post-HSCT2 maintenance therapy in MM patients, studies have explored the effect of maintenance therapy on auto-HSCT2 outcomes. The study of Pasvolsky et al. included a total of 522 patients receiving auto-HSCT2, of whom 342 received maintenance therapy and 180 did not (26). Maintenance treatment options included lenalidomide, pomadomide, and bortezomib. The results showed that the maintenance group had better NRM (2% vs 9.9%), PFS (27.8% vs 9.8%) and OS (54% vs 30.9%) at year 5 than the non-maintenance group. Maintenance therapy can improve the outcome of MM patients receiving auto-HSCT2, but there are no studies on AL patients and allo-HSCT2, and the existing studies are retrospective studies, so the maintenance therapy of HSCT2 is still an urgent problem to be solved.

Reportedly, the primary reason for the failure of HSCT2 is relapse. For patients who relapse after HSCT2, the prognosis is very poor. Few studies have been conducted on such patients, and there is no standard treatment.

Previously, EBMT conducted a study on the feasibility and effectiveness of allo-HSCT3 (27). A total of 45 patients received HSCT3. After allo-HSCT3, 38 patients were engrafted and 26 achieved CR or CRi. The 1-year NRM and CIR rates were 42% and 47%; the median PFS and OS were 2.5 and 4 months; and the 1-year PFS and OS rates were 11% and 20%, respectively. The results showed that patients who received HSCT3 had a poor prognosis. Shumilov et al. also studied coping measures after HSCT2 (28). Their single-center retrospective analysis included 14 patients with AML who relapsed after HSCT2, including 3 patients who received HSCT3, 4 patients who received DLIs combined with 5-azacytidin or venetoclax, and 7 patients who received chemoradiotherapy. Of the patients who received the first two treatments, six (86%) achieved CR and one (14%) achieved PR. After 10 months of follow-up, two patients still had CR, one patient had PR, and four patients died from relapse or NRM. Only one patient who received chemoradiotherapy achieved CR, and the rest died of relapse/refractory disease. Kobayashi et al. conducted a retrospective analysis of 253 patients with relapsed/refractory AL who received allo-HSCT3 (29). A total of 29 patients (11.5%) were alive at a median follow-up of 794 days, with 3-year LFS and OS rates of 9.7% and 10.9%, respectively. In addition, patients who maintained remission for ≥2 years after allo-HSCT2 had a significantly better 3-year OS than those who relapsed early (<1 year).

There is currently no effective therapeutic regimen for patients who relapse after a second transplantation. Third transplantation and targeted drugs may be the direction of the treatment. However, the prognosis of patients with HSCT3 remains poor, and further studies are needed to evaluate this.

## 5 Conclusion and future directions

HSCT is one of the most commonly used methods to treat hematological malignancies, but some patients still inevitably relapse. HSCT2 is a treatment option for patients with hematological malignancies after relapse after the first transplantation. Several studies, including NCT01666080 and NCT02333162, are still in progress. Many factors may affect the efficacy of HSCT2, including the duration of remission after the first transplant and disease status at the time of HSCT2. However, the donor source, difference at the time of HSCT2 and conditioning regime before HSCT2 have no significant effect on HSCT2. HSCT2 also poses the problem of relapses, and the prognosis of patients after relapse is extremely poor. However, there is a lack of standard treatment plans, which requires further research. However, there is still an critical question that need to be solved - all the studies mentioned above are retrospective observational studies, which causes a large selection bias in the selection of patients who received HSCT2. Therefore, more prospective studies are needed to explore whether HSCT2 is feasible and the treatment for patients who relapse after HSCT2.

## Author contributions

SY: Formal analysis, Writing – original draft, Writing – review & editing. XZ: Methodology, Supervision, Writing – review & editing. YX: Funding acquisition, Methodology, Project administration, Supervision, Writing – review & editing.
